# A Portable, Foldable Negative-Pressure Aerosol-Containment System (FNPACS) for Aerosol Control During Aerosol-Generating Procedures

**DOI:** 10.3390/bioengineering13060669

**Published:** 2026-06-09

**Authors:** Bing Rui Huang, Fatimah Ibrahim, Ina Ismiarti Shariffuddin, Puteri Ainaa S. Ibrahim, Li-Yen Chang, Karunan Joseph, Mas Sahidayana Mohktar, Noorjahan Haneem Md Hashim

**Affiliations:** 1 Department of Biomedical Engineering, Faculty of Engineering, Universiti Malaya, Kuala Lumpur 50603, Malaysia; 2 Centre for Innovation in Medical Engineering, Faculty of Engineering, Universiti Malaya, Kuala Lumpur 50603, Malaysia; 3 Department of Anaesthesiology, Faculty of Medicine, Universiti Malaya, Lembah Pantai, Kuala Lumpur 50603, Malaysia; 4 Department of Medical Microbiology, Faculty of Medicine, Universiti Malaya, Lembah Pantai, Kuala Lumpur 50603, Malaysia

**Keywords:** aerosol-generating procedures, aerosol containment, negative-pressure enclosure, HEPA filtration, infection control, portable medical device

## Abstract

Aerosol-generating procedures (AGPs) expose healthcare personnel to airborne pathogens and require portable engineering controls that can be integrated into routine clinical workflows. We developed a portable, foldable negative-pressure aerosol-containment system (FNPACS) combining adaptive fan control, an H14 high-efficiency particulate air (HEPA) filter, and a disposable metal-oxide prefilter in a mobile filtration module. Bench performance was evaluated using pressure-flow testing in accordance with National Environmental Balancing Bureau (NEBB) procedures and International Organization for Standardization (ISO) 14644-3, polyalphaolefin aerosol challenge testing, and smoke visualization, while an exploratory clinical study assessed environmental contamination via real-time reverse-transcription PCR (rRT-PCR) in 11 patients (31 assay analyses). Bench testing demonstrated HEPA filtration efficiencies of 99.994–99.997%, stable negative-pressure generation across fan duty cycles, no detectable downstream breakthrough beyond the HEPA filter under the tested conditions, and effective inward airflow on smoke testing. A Lagrangian discrete phase model (DPM) particle-tracking simulation further characterized size-dependent aerosol-surrogate transport. Under HEPA-ON active-extraction conditions, 73.0–86.1% of simulated 0.3–10 µm water-equivalent particles were transported to the HEPA suction pathway, while 13.9–27.0% were deposited on internal wall surfaces. In the clinical evaluation, SARS-CoV-2 RNA detection on environmental swabs was limited and predominantly low level. The clearest reproducible signal occurred on the top interior surface under HEPA-OFF conditions, whereas HEPA-ON detections were isolated or presumptive high-Ct signals without reproducible confirmation. These findings provide preliminary engineering and usability support for FNPACS as a feasible near-source aerosol-control platform for AGPs. The patient swab component should be interpreted as an exploratory, proof-of-concept assessment rather than confirmatory evidence of clinical containment efficiency because several clinical cases had non-supportive patient-related controls and were therefore not used in the primary containment interpretation.

## 1. Introduction

During the COVID-19 pandemic, tracheal intubation—one of the highest-risk aerosol-generating procedures (AGPs)—was associated with substantial infection risk among healthcare workers (HCWs). An international multicenter registry reported that approximately 10% of clinicians involved in tracheal intubation of patients with COVID-19 developed SARS-CoV-2 infection or required self-isolation, underscoring the need for protective strategies beyond personal protective equipment (PPE) alone [1]. This concern is consistent with current evidence that respiratory viruses can be transmitted through aerosols that remain suspended and are transported by indoor airflow, contributing to short- and longer-range exposure risk in healthcare settings [2,3].

One early innovation was the “aerosol box,” a transparent plexiglass enclosure placed over the patient’s head and shoulders during intubation [4]. This simple barrier device, typically incorporating arm ports for the operator, was rapidly adopted during the pandemic as an additional layer of protection. However, subsequent evaluations highlighted important limitations of passive enclosures. Their rigid structure and restricted arm ports could impair hand movement, hinder airway access and visualization, and still permit aerosol escape into the surrounding environment [5]. Broader analyses of barrier devices further emphasized that performance depends not only on physical shielding but also on geometry, airflow behavior, and workflow integration [6].

A growing body of simulation evidence also suggests that aerosol boxes may compromise airway management. Manikin studies reported longer intubation times, more frequent procedural breaches, and, in some settings, reduced first-pass success when barrier enclosures were used [7,8]. A scoping review similarly concluded that barrier enclosures may impede airway management and should therefore be used cautiously [7]. These concerns are clinically important because even modest delays in securing the airway may worsen patient hypoxemia.

Passive aerosol boxes are also limited in their ability to contain fine aerosols, particularly because infectious respiratory aerosols span a broad particle-size range and smaller particles may remain airborne and follow local airflow patterns [9]. Tracer and simulation studies showed that although such devices may intercept some large droplets, aerosolized particles can escape through open gaps and arm ports, and clinician exposure may remain substantial or even increase under some conditions [10]. Experimental comparisons of barrier devices further showed that more enclosed configurations retained aerosols more consistently, whereas active aerosol evacuation was required to reduce retained aerosol burden efficiently [11]. These concerns were reinforced by the U.S. Food and Drug Administration, which warned in August 2020 that barrier enclosures without negative pressure or suction may increase risk to patients and healthcare providers [12]. Simulation data also suggest that aerosol boxes reduce aerosol exposure reliably only in depressurized rooms, reinforcing that passive barriers are not robust substitutes for engineered airflow control in standard clinical environments [13].

Human factors and user acceptance proved to be additional challenges. Clinicians frequently reported that aerosol boxes hampered performance and comfort. In a retrospective clinical study from Mexico, 83.3% of surveyed anesthesiologists reported significant limitations in airway-device manipulation, device migration occurred in 91.6% of cases, 22.2% of cases required box removal, and only 75% would recommend continued use [14]. These findings suggest that any protective intervention is unlikely to be adopted in routine practice if it compromises procedural safety, effectiveness, or operator comfort.

To address these shortcomings, attention shifted toward negative-pressure barrier enclosures with active suction and filtration. This approach, inspired by hospital negative-pressure isolation rooms, draws air out of the enclosure through a high-efficiency particulate air (HEPA) filter so that aerosolized particles are captured rather than released into the room [15]. Several groups reported portable systems that use wall suction or vacuum assistance to generate inward airflow and route exhaust through HEPA filtration [15,16,17,18]. Related optimization studies further showed that suction strength, hood geometry, aerodynamic cap or hood design, and controlled inflow/filtration pathways are critical determinants of effective containment [19,20,21]. Collectively, these studies support the feasibility of compact bedside source-control devices and suggest that suction-assisted designs can reduce aerosol escape more effectively than passive enclosures alone.

Despite this progress, important gaps remain. Many published evaluations rely on manikin or tracer-particle surrogates rather than patient-derived microbiological measurements. In addition, comparatively few systems combine operational-range performance verification with usability-driven design refinements intended to support routine clinical deployment. Some negative-pressure enclosures have also introduced sloped viewing panels to improve airway access and visualization while preserving enclosure function [5]. These considerations motivated the development of a device that aimed to balance containment performance with practical airway access, portability, and bedside usability.

Motivated by these considerations, we developed FNPACS as a novel solution for infection control during AGPs. FNPACS is a lightweight, collapsible negative-pressure hood that envelops the patient’s head and neck and uses an integrated high-flow suction unit with HEPA filtration to actively remove aerosols. The device underwent third-party electromagnetic compatibility testing by SIRIM and was found to be compliant with applicable International Electrotechnical Commission (IEC) 60601-1-2 [22] requirements. In this study, we evaluate FNPACS from engineering, microbiological, and clinical perspectives. We characterize its airflow and filtration performance in the laboratory, evaluate preliminary aerosol-control behavior under bench conditions, and examine its usability in a simulated clinical environment. These evaluations are intended to provide early engineering and proof-of-concept clinical evidence for the design of FNPACS, while identifying limitations that should be addressed in future validation studies.

## 2. Materials and Methods

### 2.1. Design and Simulation

**Figure 1:**
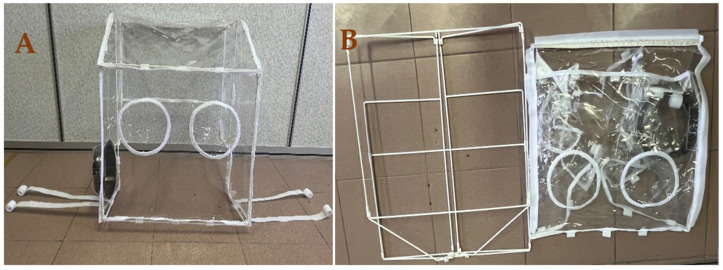
FNPACS enclosure—expanded and foldable states. (**A**) Expanded operating configuration. The transparent polypropylene hood is supported by a collapsible metal frame, with two circular glove-port sleeves on the working face and a side interface for the suction/HEPA connection. The straps along the lower edge are tie-downs to secure the enclosure to operating-table rails, preventing device migration and maintaining the chest-drape seal under negative pressure; they are quick-release to allow immediate lift-off in an emergency. (**B**) Folded (state 1) for flat-pack storage/transport. The frame collapses along its hinge joints and the soft shell lays flat, with glove ports and access panels visible on the folded cover—reflecting the ≈1 kg, flat-packable design that stows into a single compartment for rapid bedside deployment.

FNPACS was developed as a portable, transparent enclosure to surround a patient’s head and upper torso during airway procedures (Figure 1). The design builds on earlier personal containment devices (PCDs) used during COVID-19 intubations. The enclosure is a lightweight, transparent unit made of flexible polypropylene supported by a collapsible metal frame. Weighing approximately 1 kg, it is shipped flat-packed and can be quickly folded into a compact form for easy storage and transport. Its design allows rapid bedside setup and removal without obstructing emergency access. When assembled, it forms a semi-sealed hood with dimensions of approximately 50 cm (height) × 45 cm (width) × 40 cm (depth), sufficient to accommodate an adult patient’s head and neck. The front panel is equipped with two operator access ports, which are designed as sealed interfaces with self-closing silicone diaphragms to reduce the risk of leakage during procedures. Additional sealed side ports are provided for the introduction of auxiliary equipment, such as suction catheters or endoscopic tools, while preserving containment as much as possible. 

FNPACS features an active filtration unit mounted on the enclosure to generate continuous negative-pressure airflow. This unit consists of an H14-grade HEPA filter cartridge (99.995% retention at 0.3 µm) in series with a brushless DC fan. The fan draws air out of the enclosure through the HEPA filter, creating a slight vacuum inside so that any leakage is directed inward rather than outward. The suction outlet position was selected as a practical compromise between airflow extraction and clinical usability. It was placed away from the front working face to preserve airway visualization, hand-port access, assistant access, and rapid emergency lift-off of the enclosure. This arrangement was intended to draw air from the patient-facing working volume toward the HEPA pathway while keeping the operator’s viewing and working zones clear. The present study evaluated this selected outlet configuration and did not perform a full parametric optimization of outlet location. The prefilter was standardized as a titanium-dioxide (TiO_2_)-coated nonwoven prefilter intended to intercept larger and agglomerated particles and slow HEPA loading. Fan speed is adjustable from 0% to 100% pulse-width modulation duty cycle, allowing control of extraction airflow and internal negative pressure. Sensors are incorporated to monitor system performance, including a digital differential-pressure sensor across the HEPA filter and an internal pressure transducer to track enclosure pressure relative to ambient. In the current prototype, fan-speed adjustment was based primarily on the internal negative-pressure condition rather than real-time particle concentration. This pressure-based control approach was selected because a core containment objective was to maintain inward leakage and reduce the risk of aerosol escape from the enclosure. Real-time particle counting may be considered in future iterations as a supplementary monitoring tool for aerosol clearance and fan-response optimization, but it was not used as the primary control input in this prototype because particle counts are non-specific, sensitive to sensor position and background particles, and do not directly distinguish infectious viral aerosols from non-biological particles. These sensors provide real-time feedback on negative-pressure levels and filter loading. For safety, the system includes audible alarms if negative pressure is lost or if filter flow drops below a threshold. The device was developed as a medical engineering prototype for bedside aerosol-containment use.

To characterize internal flow dynamics and optimize the design, a computational fluid dynamics (CFD) simulation was performed using ANSYS Fluent (v2022 R1, ANSYS Inc., Canonsburg, PA, USA), consistent with recent CFD-informed approaches for optimizing respiratory barrier enclosures used during aerosol-generating procedures [21]. The 3D CAD model of the enclosure, including a manikin head and torso to represent a patient, was meshed with approximately 6.7 million elements after a grid-independence test (element size ~5 mm) confirmed <2% variation in key flow metrics. The shear stress transport (SST) k–ω turbulence model was employed for accurate resolution of near-wall flow and localized recirculation, consistent with prior studies of negative-pressure hoods. Realistic boundary conditions were applied to simulate a patient expiratory event together with active suction. A transient volumetric flow boundary at the manikin mouth simulated a cough sequence consisting of five expiratory events over approximately 5 s, with peak flow reaching ~10 L/s based on published cough airflow profiles. The fan-driven suction was modeled as an outlet at the HEPA filter interface with a fixed negative gauge pressure of −10 Pa, producing a steady extraction flow. Small gaps or inlet vents in the enclosure were assigned as openings at ambient pressure (0 Pa) to permit make-up air ingress. All solid surfaces, including enclosure walls and patient surfaces, were treated with no-slip wall conditions. Air was assumed incompressible at 20 °C, and gravity and buoyancy effects were included to account for expected thermal currents associated with patient breathing. To characterize aerosol-surrogate transport beyond the continuous airflow field, a Lagrangian discrete phase model (DPM) particle-tracking simulation was performed in ANSYS Fluent using the same enclosure geometry and active extraction configuration. Spherical water-equivalent inert particles with diameters of 0.3, 1, 5, and 10 µm were released from the mouth opening to represent droplets and submicron aerosol surrogates across a particle-size range relevant to infectious respiratory aerosols [9]. For each particle size, 1000 particle streams were tracked. The particle density was set to approximately 1000 kg/m^3^. Particle tracking was performed under HEPA-ON active negative-pressure conditions. The HEPA suction outlet boundary was classified as the HEPA pathway; particles leaving through this outlet were recorded by Fluent as escaped at the outlet boundary and were therefore interpreted as transported to the HEPA pathway rather than environmental escape. Particles trapped on the enclosure wall boundaries were classified as internal wall deposition. Particle trajectories were colored by residence time, and final particle outcomes were summarized as the percentage transported to the HEPA pathway or deposited on internal wall surfaces. The DPM analysis was interpreted as a computational estimate of particle transport and deposition behavior, not as a direct measurement of infectious viral capture.

**Figure 2:**
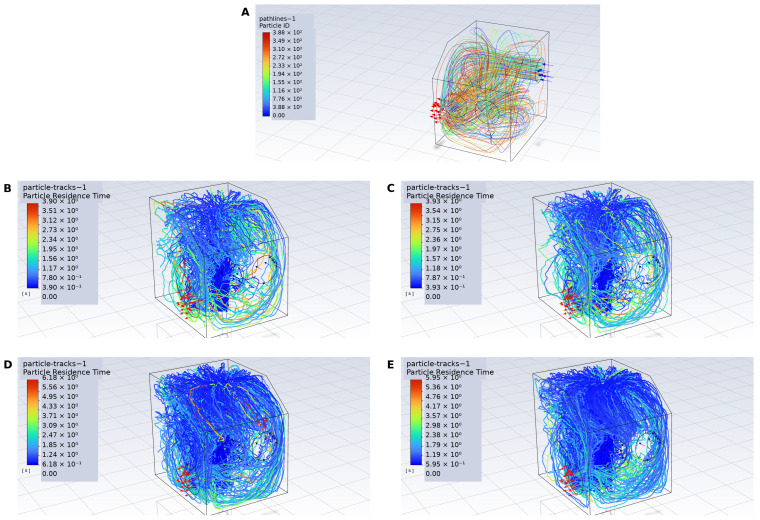
CFD airflow and DPM particle-tracking simulation of aerosol transport inside FNPACS under HEPA-ON active negative-pressure conditions. (**A**) Airflow pathlines showing directed entrainment from the patient working volume toward the HEPA suction outlet. Localized recirculation was present within the enclosure but remained contained and downstream-biased relative to the overall flow direction. (**B**–**E**) Representative Lagrangian DPM particle trajectories for 0.3 µm, 1 µm, 5 µm, and 10 µm water-equivalent particles released from the mouth opening. Particle trajectories are colored by residence time. Particles leaving through the HEPA suction outlet were classified as transported to the HEPA pathway, while particles trapped on wall boundaries were classified as internal wall deposition. The DPM results characterize particle-level transport behavior under the active extraction condition.

CFD post-processing showed a dominant inward through-flow from ambient ingress regions toward the HEPA suction outlet. As expected for a partially enclosed negative-pressure hood, localized recirculation structures were present within the enclosure, particularly near geometric obstructions and in the upper canopy, but these remained contained and downstream-biased relative to the overall flow direction. Importantly, the computed flow field did not show a persistent outward leakage pathway directed toward the access openings. The DPM particle-tracking analysis further extended this airflow interpretation by showing particle-level transport of aerosol surrogates toward the HEPA pathway and internal wall surfaces. The CFD and DPM findings were therefore used together to characterize the enclosure airflow path and particle-transport behavior, while aerosol containment performance was evaluated separately via bench PAO challenge and smoke-visualization testing. The simulated airflow field and representative DPM particle trajectories are shown in Figure 2.

CFD was used to characterize the direction of induced airflow inside the enclosure and to determine whether the dominant flow pathway was oriented toward the HEPA suction outlet. The simulation outputs included airflow pathlines, velocity fields, pressure distribution, and particle-transport behavior from the DPM analysis. These computational results were interpreted together with the bench smoke-visualization observations to assess whether the predicted inward-directed flow pattern was qualitatively reproduced under physical testing conditions.

### 2.2. Bench Testing and Performance of FNPACS

Laboratory testing of the FNPACS prototype was conducted to characterize airflow, pressure performance, filtration performance, and potential leakage in accordance with cleanroom and containment standards. Airflow and pressure measurements were performed based on ISO 14644-3:2019 [23] and National Environmental Balancing Bureau (NEBB) [24] procedural standards for controlled environments. All tests were carried out in a temperature-controlled laboratory (22 ± 2 °C; relative humidity ~50%), with the enclosure positioned over a supine manikin to simulate patient positioning during use. The active filtration unit was operated at fan-speed settings from 10% to 90%.

Air inflow velocity was measured using a TSI 9515 hot-wire anemometer positioned at the center of the prefilter opening. At each fan-speed setting, the differential pressure across the HEPA filter was recorded using the installed pressure sensor. The corresponding velocity–pressure relationship is summarized in Table 1.

**Figure 3:**
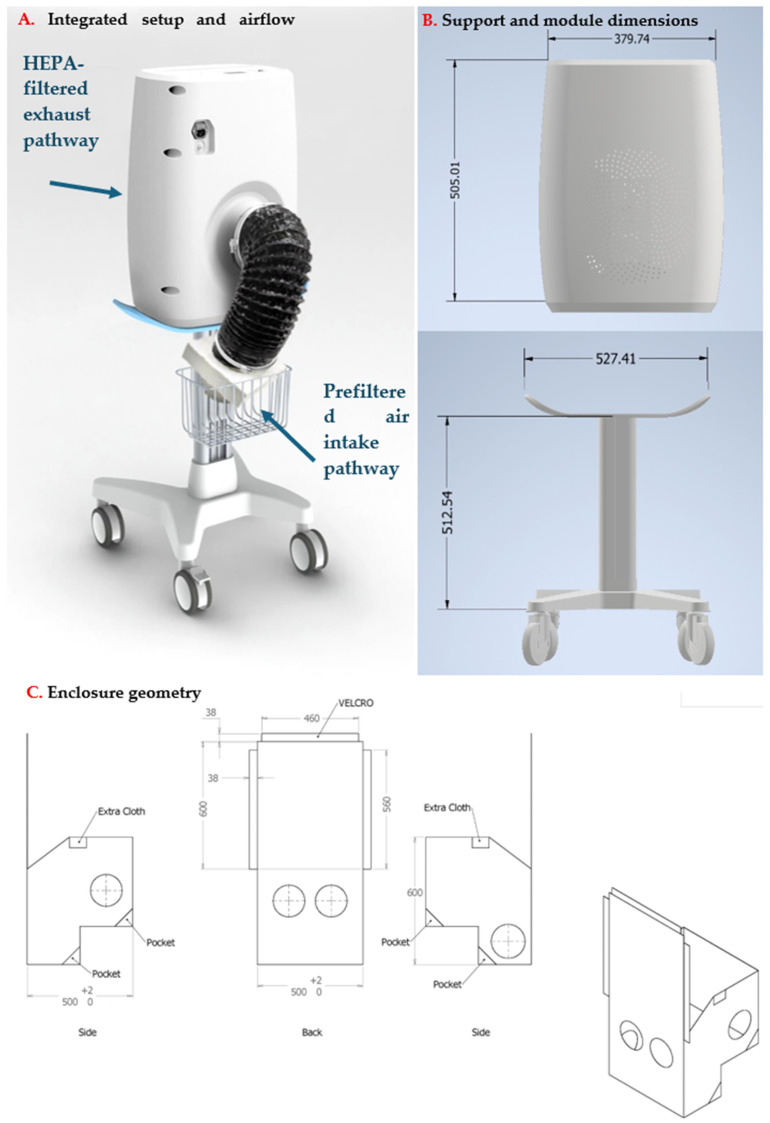
Technical schematic of the FNPACS system configuration, dimensions, and airflow pathways. (**A**) Integrated FNPACS setup showing the mobile support structure, filtration module, flexible hose connection, prefiltered air-intake pathway, and HEPA-filtered exhaust pathway. The airflow route indicates how air is drawn through the prefilter and directed through the HEPA filtration pathway before exhaust. (**B**) Support and filtration-module dimensions, showing the main dimensional features of the mobile support and upper filtration housing. (**C**) Enclosure geometry and foldable chamber layout, showing the main side and rear panel dimensions, access-port positions, pocket/cloth interfaces, and assembled enclosure configuration.

Containment leakage assessment was performed using both qualitative smoke visualization and quantitative aerosol measurements. In the smoke test, a commercial fog generator (producing sub-micron glycerin-based fog) was placed inside the enclosure and activated for several seconds to fill the interior with visible smoke. The FNPACS fan was then run to create negative pressure. A high-resolution video camera was used to record smoke movement. Colored illumination was used to enhance visualization of the smoke plume direction during image and video recording, consistent with recent optical flow-visualization approaches for assessing airflow dynamics in negative-pressure isolation chambers [25]. The smoke uniformly flowed toward the HEPA intake, and upon deactivation of the fogger, the internal smoke rapidly cleared, confirming robust ventilation of the chamber. Figure 3 schematically illustrates the technical configuration of FNPACS, including the foldable enclosure geometry, support and filtration-module dimensions, prefiltered air-intake pathway, HEPA-filtered exhaust pathway, and the overall airflow route through the negative-pressure system. The laboratory smoke-visualization test further showed that smoke-retention performance passed at fan-speed settings of 70% and above. In the absence of a patient-simulated occupant inside the enclosure, running the fan at 70% speed successfully and rapidly expelled the visible smoke. When a patient-simulated occupant was present, smoke appeared to be drawn more directly toward the HEPA intake, likely because the occupant reduced the effective open area and altered the local flow path. This observation was based on visual review of the smoke-visualization recording and was not separately quantified by local velocity mapping. Therefore, it was treated as a qualitative observation and was not used to recommend a lower operating fan-speed setting.

For quantitative aerosol leakage testing, we adopted the polyalphaolefin (PAO) aerosol challenge method commonly used for HEPA filter integrity testing. A PAO aerosol generator (ATI TDA-4B, Air Techniques International, Owings Mills, MD, USA.) was used to introduce a polydisperse aerosol of PAO droplets (~0.3 µm mass median diameter) into the enclosure upstream of the HEPA filter. The aerosol was generated at a concentration of ~24 µg/L, within the 20–30 µg/L range recommended by ISO 14644-3 [23], to rigorously challenge the system’s containment. While the aerosol was being drawn through the filter, an aerosol photometer probe (ATI 2i Photometer, Air Techniques International, Owings Mills, MD, USA) was used to scan along all seams, glove penetrations, and around the perimeter of the access panels to detect any escaping aerosol. The photometer sampling probe was held ~5 cm outside the enclosure, moving slowly along each potential leakage site, and it continuously measured the downstream aerosol concentration as a percentage of the challenge concentration. In addition, nine specific locations around the front opening (corresponding to a 3 × 3 grid covering the open panel area) were sampled for 10 s each to mimic a standardized leak-test mapping. Across all measurements, the leakage readings remained at or near the photometer’s baseline, indicating no detectable aerosol penetration outside the enclosure beyond the filter. In particular, the PAO concentration immediately outside the HEPA filter outlet remained <0.001% of the inside level, confirming the filter’s efficacy. For comparison, a control test without the enclosure showed widespread aerosol dispersal around the manikin. After each aerosol test, the enclosure interior was thoroughly purged and the HEPA filter was rescanned to ensure that no residual PAO seeped through. All sensors and instruments were calibrated, and the testing procedures were reviewed by certified cleanroom professionals for compliance with ISO/NEBB [23,24] methodologies. These results gave confidence that FNPACS can maintain negative-pressure containment with negligible leakage even under vigorous aerosol challenges.

### 2.3. User Acceptance Testing

A structured user acceptance test was conducted at Universiti Malaya Medical Centre (UMMC) to evaluate the usability, safety, and perceived clinical applicability of the FNPACS device during simulated airway-management tasks, consistent with the use of manikin-based simulation to assess the clinical usability of airway-related devices before broader clinical implementation [26]. The original usability exercise involved 49 clinicians ranging from 1st-year to 4th-year medical officers and trainees, with a roughly balanced gender distribution. Participants interacted with the device in a simulated clinical setting and completed a structured questionnaire covering 38 usability dimensions on a five-point Likert scale. In addition to the quantitative assessment, open-ended feedback was collected and categorized into three domains: features to keep, features to remove, and features to modify.

Two non-discriminating response sets consisting entirely of “strongly disagree” selections across the questionnaire were excluded, yielding 47 evaluable responses. The analyzed usability domains included usefulness, task facilitation, safety, ease of use, learnability, flexibility, physical effort, logical workflow, device dimensions, chamber opening dimensions, weight, disposal, appearance, future confidence, and frequency of intended use. Descriptive analysis was performed by summarizing dominant response categories and agreement patterns across items.

Qualitative comments were reviewed thematically to identify recurrent design strengths and priorities for refinement. Particular attention was given to recurring comments related to the HEPA filter/negative-pressure concept, portability and foldability, transparency, assembly, fastening method, hand-port ergonomics, frame stability, and access for assistants or adjunct airway devices.

### 2.4. Clinical Viral Containment Evaluation

A prospective exploratory clinical swab study was conducted to characterize the contamination patterns associated with FNPACS during coached coughing. This environmental sampling approach was informed by prior hospital-based studies showing that SARS-CoV-2 RNA can be detected in air and surface samples from patient-care environments, with contamination patterns influenced by patient factors, room airflow, and ventilation conditions [27,28]. The protocol was approved by the institutional ethics review board (MREC ID: 2021129-9971), and all participants provided written informed consent. Adults (≥18 years) hospitalized with confirmed COVID-19 who were clinically stable enough to perform coached coughing and who agreed to the use of FNPACS were eligible. Patients with hemodynamic instability, inability to cooperate with coughing instructions, or contraindications to enclosed spaces were excluded.

A total of 11 patients (P1–P11) were enrolled. For microbiological evaluation, rRT-PCR was performed in triplicate for P1–P9 and in duplicate for P10 and P11, yielding 31 assay analyses in total. Each patient underwent sequential testing under two conditions. First, an FNPACS enclosure was used without the air-exhaust motor/HEPA filter activated (B1). Second, a new FNPACS enclosure connected to the HEPA filtration system was used (B2). Under each condition, the patient was placed supine, standard physiological monitoring was applied, and the patient was instructed to cough forcefully five times into the enclosure.

**Figure 4:**
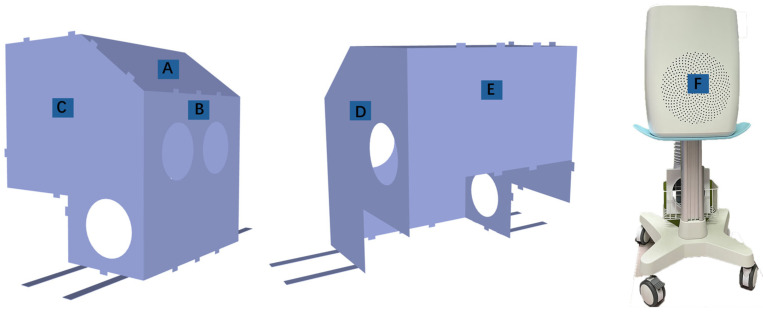
The six swab positions of the enclosure used in the clinical trial were designated as follows: A—Top surface, B—Front panel, C—Left side, D—Right side, E—Back panel, and F—Filter-outlet exterior surface (A–E: enclosure patient-facing).

Surface swab sampling followed the clinical workflow used during testing. The sampling sites were harmonized as follows: A, top surface; B, front panel; C, left side; D, right side; E, back panel; and F, filter-outlet exterior surface. The locations of the six swab sampling sites are illustrated in Figure 4. In the grouped summary, sites C, D, and E were pooled as Other Positions (C, D, E). All swabs were collected from predefined 25 cm^2^ areas using sterile swabs placed into viral transport medium, double-bagged, transported on ice, and processed in the Medical Microbiology Laboratory.

In the laboratory, viral RNA was extracted from the swab medium using a Qiagen viral RNA kit according to the manufacturer’s protocol. rRT-PCR was performed on each sample using a multiplex assay (Allplex 2019-nCoV Assay, Seegene Inc., Seoul, Republic of Korea) targeting three SARS-CoV-2 genes: the Envelope gene (E), the Nucleocapsid gene (N), and the RNA-dependent RNA polymerase/Spike gene (RdRp/S). Amplification was conducted on a Bio-Rad CFX96 real-time PCR system with the following cycling parameters: 50 °C for 20 min for reverse transcription, 95 °C for 15 min for initial denaturation, followed by 45 cycles of 95 °C for 10 s, 60 °C for 15 s and 72 °C for 10 s. For the primary interpretation of clinical containment, patient cases were considered interpretable only when the corresponding patient-related positive controls supported the presence of SARS-CoV-2 RNA during the sampling sequence. Cases with negative, unavailable, or otherwise non-supportive Petri dish and/or oral-cavity controls were classified as control-limited/inconclusive. 

### 2.5. Statistical Analysis

This study was primarily exploratory in nature and was analyzed using descriptive statistics. Continuous bench-testing variables are presented as the mean ± standard deviation (SD) where repeated measurements were available. Categorical variables are summarized as counts and percentages. For the user-acceptance assessment, questionnaire responses were analyzed descriptively using frequency distributions and agreement rates, where agreement was defined as the combined proportion of strongly agree, agree, and somewhat agree responses. For manuscript-level analysis, 49 returned questionnaires were reviewed and two non-discriminating response sets were excluded, yielding 47 valid responses for quantitative analysis.

Clinical swab results were also analyzed descriptively due to the small sample size. Accordingly, the 11 enrolled patients and 31 assay outputs were summarized using counts, proportions, and grouped site-level patterns under HEPA OFF and HEPA ON conditions. No formal hypothesis-testing framework was prespecified for the clinical analysis, and the clinical findings should therefore be interpreted as exploratory rather than confirmatory. Data tabulation and descriptive analyses were performed in Microsoft Excel. For the clinical swab dataset, results were summarized in two layers: control-valid and reproducible findings were used for the primary interpretation, whereas control-limited, isolated, or presumptive findings were described only for transparency. No clinical containment-efficiency percentage was calculated from the patient swab data.

## 3. Results

FNPACS performance was assessed using ANSYS Fluent simulation, ISO 14644-3/NEBB [23,24]-guided bench testing, and exploratory clinical evaluation. The results are presented in the following sections.

### 3.1. Engineering Simulation Results

The airflow behavior inside FNPACS was simulated using ANSYS Fluent to evaluate whether the enclosure geometry and extraction configuration could establish a directed negative-pressure flow field during a simulated cough event. The resulting streamline patterns showed that air was predominantly drawn from the ingress regions and patient working zone toward the HEPA suction outlet at the rear of the chamber. This indicates that the principal flow direction within the enclosure was aligned with the intended aerosol-removal pathway.

The simulated flow field was not completely free of recirculation. Instead, localized recirculation structures were observed in restricted regions, particularly near geometric obstructions and in the upper canopy. However, these recirculation zones remained contained within the enclosure and were downstream-biased relative to the overall flow direction. Importantly, the simulation did not show a persistent backflow pattern or an outward leakage pathway from the patient zone toward the access openings. In this respect, the CFD results support the function of the negative-pressure configuration as an active containment system rather than a passive barrier enclosure.

Turbulence kinetic energy was highest near the cough jet and in the vicinity of the suction inlet, where velocity gradients were greatest. Outside these regions, turbulence levels were lower in the peripheral working volume, suggesting that mixing was concentrated near the source and extraction regions rather than being distributed uniformly throughout the enclosure. This pattern is consistent with a localized extraction system in which the dominant mechanism is directed entrainment toward the outlet.

Overall, the CFD findings suggest that FNPACS generates a controlled inward airflow with limited, contained recirculation and a dominant transport pathway toward the HEPA unit. These simulation results were used to support design refinement of the enclosure geometry and airflow path, with the aim of improving aerosol capture and minimizing stagnant or weakly ventilated regions.

The DPM particle-tracking simulation showed size-dependent transport behavior under HEPA-ON active negative-pressure conditions, as summarized in Table 2. For 0.3 µm particles, 86.1% were transported to the HEPA pathway and 13.9% were deposited on internal wall surfaces. For 1 µm particles, the corresponding values were 84.0% and 16.0%. For 5 µm particles, 76.3% were transported to the HEPA pathway and 23.7% were deposited internally, while for 10 µm particles, 73.0% were transported to the HEPA pathway and 27.0% were deposited on internal wall surfaces. Overall, 79.9% of the 4000 tracked particles were transported to the HEPA pathway and 20.2% were deposited on internal walls. These findings indicate that smaller particles were more readily carried by the directed airflow toward the HEPA suction pathway, whereas larger particles showed a greater tendency toward internal surface deposition. No particles remained incomplete at the final tracking time in this DPM analysis. These CFD and DPM findings were qualitatively compared with bench smoke-visualization observations to determine whether the predicted inward-directed flow pattern toward the HEPA pathway was reproduced under physical testing conditions.

### 3.2. Bench Testing and Performance of FNPACS

Bench testing showed a progressive increase in inflow velocity with increasing fan-speed setting (Table 1). Inflow velocity rose from 0.18–0.20 m/s at 10% fan speed (approximately 1330 rpm) to 2.70–2.78 m/s at 90% fan speed (approximately 11,970 rpm). Over the same operating range, the differential pressure across the HEPA filter increased from −0.7 to −67 Pa, indicating a progressively greater pressure load across the filtration module as the fan speed increased. At intermediate operating points, inflow velocity and pressure response also remained stable; for example, at 50% fan speed, the measured inflow velocity was 1.14–1.18 m/s with a differential pressure of −20 Pa. Together, these findings indicate that FNPACS generated consistent inward airflow and predictable pressure performance across the tested fan-speed range.

PAO aerosol challenge testing demonstrated high filtration integrity. Under operational conditions, the calculated upstream challenge concentration was approximately 24 µg/L, while the measured leak penetration remained below the acceptance criterion of 0.01% of the upstream concentration for both the supply path (<0.006%) and the exhaust path (<0.008%). The corresponding HEPA filtration efficiency was 99.997%, supporting effective aerosol retention by the filtration module.

**Figure 5:**
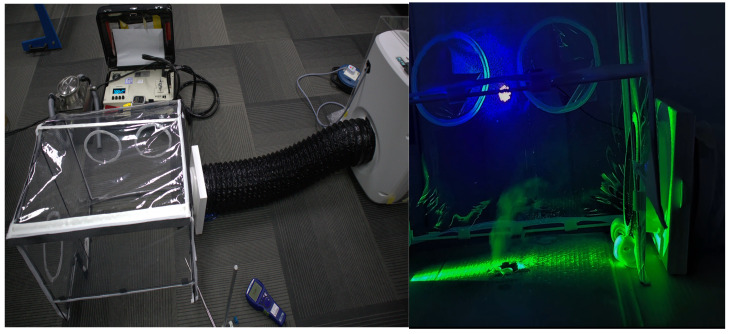
Representative smoke-visualization image of inward-directed airflow during FNPACS operation. Visible smoke was generated inside the enclosure under active extraction. The smoke plume was drawn toward the filtration intake, while no visible outward smoke escape was observed through the access ports or lower opening under the tested condition. This image provides qualitative visual support for the inward-directed airflow and negative-pressure containment behavior observed during bench testing.

Qualitative smoke visualization also supported the containment performance of the system. In the empty enclosure, visible smoke cleared rapidly at a fan setting of 70%, and smoke-retention performance was considered acceptable at fan settings of 70% and above under the laboratory test conditions. The smoke stream was directed toward the filter inlet, with no visible escape from the access ports or seams. Representative smoke-visualization imaging is shown in Figure 5. Under active extraction, the visible smoke plume generated inside the enclosure was drawn inward and directed toward the filtration intake rather than dispersing outward through the access ports or lower opening. The observed plume direction was qualitatively consistent with the CFD-predicted inward through-flow and downstream-biased transport toward the HEPA suction pathway. Therefore, the smoke-visualization results provided physical bench support for the airflow behavior predicted by the CFD analysis, while the DPM simulation further characterized particle-level transport within the enclosure. Under patient-simulation conditions, smoke movement visually appeared more directed toward the filter inlet, probably because the occupant reduced the effective open area inside the enclosure. This was a qualitative smoke-visualization observation rather than a separate velocity measurement; therefore, no lower fan-speed threshold was assigned from this finding.

### 3.3. User Acceptance Testing

**Figure 6:**
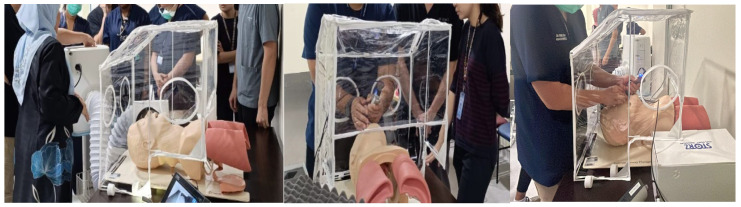
User acceptance testing of the FNPACS device by medical officers and trainees at Universiti Malaya Medical Centre (UMMC). The original usability exercise involved 49 clinicians and evaluated 38 usability dimensions using a five-point Likert scale, together with open-ended feedback on features to keep, remove, and modify. In total, 47 evaluable responses were retained after exclusion of two non-discriminating response sets.

A hands-on usability evaluation of the FNPACS device was conducted at Universiti Malaya Medical Centre (UMMC). A usability evaluation was completed by 49 clinicians. After the exclusion of two uniform response sets, 47 responses were included in the analysis. An overview of the user acceptance testing dataset is presented in Figure 6. Two non-discriminating response sets consisting entirely of “strongly disagree” selections across the questionnaire were excluded, yielding 47 evaluable responses.

Overall acceptance was high. Using the cleaned dataset (*n* = 47), the mean agreement across the scored usability items was 86.0%, where agreement was defined as the combined proportion of strongly agree, agree, and somewhat agree responses. The results of the main questionnaire items are summarized in Table 3. The strongest-performing domains were safety, learnability, ease of use, appearance, and weight. In particular, “allows me to ensure my safety” achieved 97.9% agreement, “is easy to use (setting control panel)” achieved 95.7% agreement, “I learnt to use it quickly” achieved 97.9% agreement, “I learnt to use it easily” achieved 95.7% agreement, “I easily remember how to use it” achieved 97.9% agreement, and “allows me to complete tasks in a logical sequence” achieved 91.5% agreement. These findings indicate that the system was generally perceived as safe, easy to learn, and compatible with a logical procedural workflow.

Physical design attributes were also rated favorably. Agreement levels with “the dimensions of the device” and “the dimensions of the foldable intubation chamber” were 76.6% and 76.6%, respectively, while “the opening dimensions of the intubation chamber are reasonable for intubation procedure” achieved 78.7% agreement. Device- and chamber-weight items were rated positively, with agreement levels of 91.5% and 95.7%, respectively. “the foldable intubation chamber is easy to dispose” reached 93.6% agreement, and “the appearance of the device is reasonable” reached 97.9% agreement. Together, these results support the perception that the foldable chamber is lightweight, visually acceptable, and broadly practical for clinical handling.

Not all dimensions were rated equally strongly. Domains related to speed, task control, recovery from mistakes, frequent future use, and adjustability showed more moderate response profiles. “Allows me to complete my tasks quickly” had 59.6% agreement, “allows me better control on performing my tasks” achieved 66.0% agreement, and “allows me to recover from mistakes quickly and easily” achieved 74.5% agreement. Likewise, “I will like to use it frequently” had 68.1% agreement, with 25.5% of respondents selecting a neutral response, while “It will be easy to adjust it to perform my work” reached 83.0% agreement, with 14.9% neutral responses. These results suggest that although the device was generally well accepted, routine workflow integration and adjustability remain important targets for refinement.

The qualitative feedback broadly matched the quantitative results. Users most often highlighted the HEPA filter, the negative-pressure mechanism, the foldable design, the lightweight structure, the transparency of the enclosure, and ease of assembly as key strengths. The most common suggestions for improvement were to replace or reinforce the Velcro-based fastening system, enlarge or reshape the hand ports, improve frame stability, add access points for assistants, and further reduce the frame weight by using lighter materials. These points were in line with the original usability findings, which showed that the system was generally well accepted in clinical use while also pointing to clear priorities for the next design iteration. Overall, the user acceptance results suggest that FNPACS was viewed as safe, portable, and easy to learn, and they provide practical guidance for further ergonomic improvement.

### 3.4. Clinical Evaluation Outcomes

The exploratory clinical dataset included 11 patients (P1–P11). For microbiological evaluation, rRT-PCR was performed in triplicate for P1–P9 and in duplicate for P10 and P11, yielding 31 assay results in total. These 31 results do not represent 31 independent patient trials but instead reflect repeated assay outputs from 11 patient cases. The patient-level clinical trial outcomes across the sampling positions are summarized in Table 4. Under the study interpretation criteria, a sample was regarded as truly positive only if all replicates were positive. A signal detected in only one replicate was not considered definitive, and E-gene-only detection was classified as presumptive. Based on these criteria, P1–P8 were classified as control-limited/inconclusive because the Petri dish and/or oral-cavity controls were negative, unavailable, or otherwise non-supportive. These cases were not included in the primary interpretation of containment performance. They are retained in Table 4 to show the complete clinical record, but isolated high-Ct or presumptive signals from these cases were interpreted descriptively only and were not taken as evidence of either device efficacy or device failure.

At the individual patient level, P1 showed no environmental detection in any assay output; only the assay positive control was positive. Among P2–P8, several weak or isolated signals were recorded, but these observations were treated as descriptive findings only because the patient-related positive controls did not support reliable interpretation. P2 showed an isolated front-panel signal under B2 conditions with an N-gene Ct of 37.79. P4 showed presumptive front-panel signals under both B1 and B2 conditions, with Ct values of 37.82 and 37.81, respectively. An additional isolated front-panel signal was detected under B2 conditions with a Ct of 36.3. P5 showed a presumptive front-panel signal under B1 conditions with a Ct of 37.99. P6 showed an isolated front-panel signal under B2 conditions with an RdRp/S-gene Ct of 38.57. Since these cases lacked supportive patient-related positive controls, they were not used in the primary containment interpretation.

For P9, one replicate from the front panel under B2 conditions was positive with a high Ct value of 38.34. However, the Petri dish control was negative, and only the oral cavity control was confirmed positive. This case was therefore treated as an isolated signal rather than a confirmed contamination event.

P10 provided the clearest evidence of reproducible SARS-CoV-2 RNA detection within duplicate analyses of an environmental sample in the study. The top surface under B1 conditions was positive in repeated duplicate analyses. In one analysis, the E gene and N gene were detected at Ct 36.34 and 37.77, respectively. In the subsequent duplicate analyses, both targets remained positive at Ct 37.74 and 37.51. Both patient-related positive controls were also positive in this case, indicating that sampling and processing were valid. Under B2 conditions, by contrast, the filter-outlet exterior surface showed only one presumptive E-gene-only signal at Ct 38.21, and this was not reproduced in the next duplicate analyses. This case therefore supports reproducible top-surface contamination under B1 conditions, whereas the outlet-associated signal under B2 conditions remained isolated.

For P11, no environmental swab was confirmed positive at the case level, although the oral cavity control was positive. Individual assay outputs did show presumptive signals at the front panel under B2 and B1 conditions (Ct 39.37 and 37.79, respectively). A presumptive signal was also detected at the filter-outlet exterior surface control swab under B2 conditions (Ct 37.75) As this control sample was obtained before placement of the FNPACS over the patient, the detected signal cannot be attributed to be patient-derived and may instead reflect background contamination. Furthermore, the signal was not reproducible on repeat testing. Overall, P11 is better regarded as showing isolated presumptive signals rather than confirmed positive results.

When results were grouped by surface location, the most reproducible signal was seen at site A (Top surface) under B1 conditions, mainly because of P10. Site B (Front panel) accounted for most of the isolated or presumptive high-Ct detections, including P2, P4, P5, P6, P9, and P11. The pooled peripheral sites C/D/E remained negative throughout. Site F (filter-outlet exterior surface) showed only isolated or presumptive signals and no reproducible confirmed positivity.

Overall, the clinical swab findings support a cautious interpretation. The clearest reproducible contamination signal was found at the top surface when the device was used without active extraction. Under B2 conditions, the detected signals were few, weak, and not reproducible, and they were seen mainly at the front panel or the filter-outlet exterior surface. These findings are better understood as exploratory evidence of where contamination may localize, rather than definitive proof of complete viral filtration during patient use.

**Figure 7:**
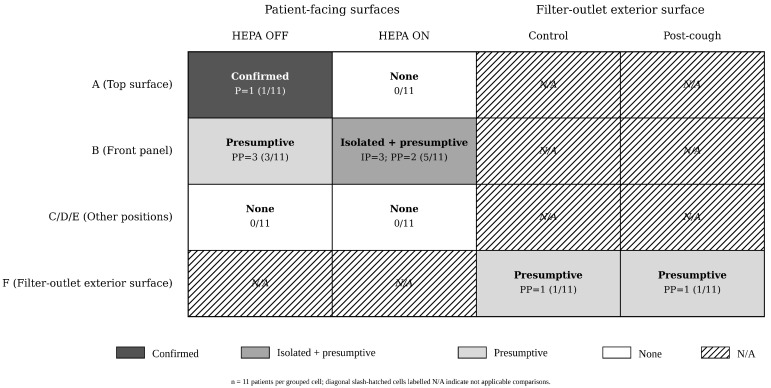
Summary matrix of exploratory rRT-PCR findings by grouped site and condition. The matrix summarizes patient-level grouped findings across swab sites and study conditions (*n* = 11 patients per grouped cell). Patient-facing surfaces include A (top surface), B (front panel), and pooled C/D/E (other positions), each compared under HEPA OFF and HEPA ON conditions. Site F represents the filter-outlet exterior surface and is summarized separately using control and post-cough swabs collected under B2 conditions. Cell shading indicates the highest level of evidence present within each grouped cell (confirmed, isolated + presumptive, presumptive, or none), while diagonal slash-hatched cells labelled N/A indicate not applicable comparisons comparisons. The clearest reproducible signal was observed at the top surface under HEPA-OFF conditions, whereas detections under B2 conditions were limited to weak, isolated, or presumptive findings, mainly at the front panel or filter-outlet exterior surface.

The grouped-site summary shown in Figure 7 should be interpreted in the context of assay reproducibility and control validity. Site A (top surface) showed the clearest reproducible contamination signal under B1 conditions, driven by P10, in which the top-surface swab was positive in repeated duplicate analyses (Ct 36.34/37.77 and 37.74/37.51). By contrast, site B (Front panel) accounted for most of the weak or isolated detections, including P2 (Ct 37.79), P4 (Ct 37.82 and 37.81), P5 (Ct 37.99), P6 (Ct 38.57), P9 (Ct 38.34), and P11 (Ct 37.79 and 39.37). However, most of these signals either lacked reproducibility across replicates or occurred in cases with non-supportive patient-related positive controls and were therefore not treated as confirmed positive environmental findings. Site F (filter-outlet exterior surface) showed only isolated or presumptively positive signals, including the non-reproducible P10 signal (Ct 38.21) and the presumptive signal detected in the P11 pre-use control swab (Ct 37.75), which was not considered a confirmed positive. No reproducible confirmed positive signal was identified at the grouped peripheral positions C, D, and E. Collectively, these patterns suggest that when viral material was detected, it was localized mainly to the top or front patient-facing surfaces. After separating control-limited cases from control-valid findings, the main interpretable clinical observation was the reproducible top-surface signal under HEPA-OFF in P10. Other high-Ct, isolated, or presumptive findings were retained for completeness but were not used to estimate clinical containment efficiency.

## 4. Discussion

This study provides preliminary engineering, bench-testing, and usability evidence to support further development of FNPACS as a bedside aerosol-control platform for aerosol-generating procedures. The bench data showed stable airflow generation, effective filtration, and no detectable downstream breakthrough during PAO challenge. By contrast, the patient swab data were more informative about contamination localization than about absolute clinical containment efficiency. The clinical component should therefore be interpreted as exploratory and proof-of-concept, reflecting contamination patterns and device behavior under the tested conditions rather than definitive clinical efficacy.

### 4.1. Engineering Innovation and Aerosol Containment Performance

A primary contribution of this work is the engineering design of FNPACS as an integrated containment platform rather than a passive barrier alone. Beyond aerosol containment itself, the innovation lies in the combination of a foldable, lightweight, bedside-deployable enclosure and a dedicated negative-pressure filtration module specifically developed for this hood. Unlike rigid barrier systems, the collapsible, approximately 1 kg design supports flat-pack storage, rapid bedside setup, transportability, and practical post-use handling. In parallel, the enclosure was paired with a purpose-built filtration unit, HEPA filter, prefilter, pressure sensing, and alarm functions, enabling controlled inward airflow and active source-directed aerosol capture. This design rationale is consistent with evidence that infectious respiratory aerosols can remain suspended, follow indoor airflow patterns, and span a broad particle-size range relevant to infection-control interventions [2,3,9].

FNPACS differs from a passive barrier because its performance depends on maintaining a directional through-flow rather than simply placing a shield around the patient. The progressive rise in inflow velocity and pressure drop with fan speed suggests that the enclosure and filtration unit behaved as an integrated flow system: stronger extraction increased entrainment toward the outlet and likely reduced aerosol residence time within the working volume. The CFD results are helpful in this context not because they prove particle capture directly but because they show why the bench findings are plausible. The simulated recirculation remained localized and downstream-biased instead of forming a persistent outward leak path. The DPM particle-tracking results further support this interpretation by extending the analysis from bulk airflow direction to particle-level transport behavior. While streamline plots show the direction of the continuous airflow field, DPM tracking provides a closer computational representation of how droplets and submicron aerosol surrogates move within the enclosure. Under HEPA-ON conditions, most simulated particles were transported to the HEPA pathway, with transport fractions ranging from 73.0% to 86.1% across the tested 0.3–10 µm particle sizes. The remaining particles deposited on internal wall surfaces, with deposition increasing as particle size increased. This size-dependent pattern is consistent with the expected behavior that smaller particles more closely follow airflow pathways, whereas larger particles are more influenced by inertia and surface deposition. These results support the engineering rationale that FNPACS functions through active source-directed extraction rather than passive shielding alone. Nevertheless, the DPM results should be interpreted as a computational estimate rather than a direct measurement of infectious aerosol capture. Particle evaporation, particle charge, agglomeration, patient-specific cough variability, and viral viability were not modeled. The current outlet position should also not be regarded as a fully optimized geometry. Outlet location is expected to influence recirculation, local clearance, pressure drop, and measured inflow velocity. In this prototype, the outlet location was chosen to balance aerosol extraction with practical clinical constraints, including airway visibility, hand access, assistant access, emergency removal, and foldability. Future design work should therefore include parametric CFD and bench testing of alternative outlet positions and extraction geometries to identify configurations that improve clearance while preserving operator workflow. In practical terms, the protective value of the device appears to result from airflow control and source-directed removal, which is precisely the limitation of passive boxes. The qualitative smoke-visualization observation further supported this interpretation by showing that the visible plume was drawn inward toward the filtration intake during active extraction. The agreement between the CFD-predicted airflow pattern, the DPM particle-transport behavior, and the smoke-visualization observation strengthens the engineering evidence that FNPACS establishes inward-directed flow rather than functioning solely as a passive barrier. Comparable patient-covering negative-pressure boxes have also been reported in other aerosol-generating procedures, such as esophagogastroduodenoscopy, supporting the broader applicability of local negative-pressure containment concepts beyond tracheal intubation [29].

The patient swab study is most informative when interpreted as a localization study rather than as a binary success–-failure test. The reproducible HEPA-OFF signal on the top surface in P10 suggests that exhaled material tends to deposit on patient-facing internal surfaces when active extraction is absent, while the lack of reproducibly confirmed downstream breakthrough under HEPA-ON conditions indicates that any residual leakage, if present, was low and inconsistent under the tested conditions. The predominance of high-Ct, non-reproducible signals under B2 conditions is also compatible with contamination near the detection limit or with variability introduced by patient-specific coughing dynamics and surface sampling. The number of control-limited cases should be noted. Several factors may explain the non-supportive Petri dish and/or oral-cavity controls. Viral shedding during coached coughing can vary with disease stage, cough effort, and sampling time. Droplet deposition on a Petri dish may also be uneven, especially when the collection area is small and the cough plume is not directed uniformly. In addition, surface-swab recovery can be affected by swabbing pressure, surface wetness, transport conditions, and the low amount of recoverable RNA on environmental surfaces. PCR inhibition is another possible analytical issue in environmental swab studies, although an internal positive control was included to monitor assay performance. For these reasons, control-limited cases were treated conservatively and excluded from the primary containment interpretation. Therefore, the clinical swab data should not be used to calculate a quantitative viral containment efficiency. Their main value is to show where detectable viral RNA may localize during use and to guide the design of larger, control-valid clinical studies.

This pattern is more informative than any single Ct value. It is consistent with the intended role of active extraction: not to make the patient zone sterile, but to reduce uncontrolled outward escape and shift any detectable contamination toward internal, more controllable surfaces. This interpretation is consistent with the engineering rationale of the device and with prior literature showing that barrier-plus-extraction strategies reduce exposure more effectively than passive boxes alone [30,31]. FNPACS is therefore better viewed as a risk-reduction device that complements PPE and room-level precautions than as a standalone substitute for them. The localization of detected viral RNA mainly on the top and front patient-facing surfaces should not be interpreted as evidence that FNPACS performs only marginally better than passive enclosures. In a passive enclosure, aerosols may be physically intercepted or temporarily retained, but leakage direction is not actively controlled and unfiltered air may still escape through access openings, gaps, or during enclosure removal. By contrast, the active function of FNPACS is to maintain a negative-pressure environment, draw air toward the HEPA pathway, and filter extracted air before release. Therefore, internal surface detection in this exploratory swab study is better interpreted as evidence of where viral material may deposit within the enclosure, rather than as proof of equivalence to passive barrier devices.

### 4.2. Comparison with Passive Boxes and Other Barrier Systems

FNPACS was designed to address several limitations of earlier passive aerosol-containment devices. During the COVID-19 pandemic, improvised acrylic “aerosol boxes” gained popularity as a quick protective measure during intubations [32]. While these boxes provided a physical barrier against large droplets, they often lacked any active air handling. Research soon highlighted their limitations: a transparent box with arm holes cannot fully prevent aerosol dispersion into the room. In fact, particles can escape through the open sides and arm ports, accumulating both inside and around the patient. Chang et al. (2024) noted that a simple acrylic shield without suction failed to completely block the transmission of droplets and aerosols, primarily because it was not airtight and allowed leakage around the patient’s torso and bed [30]. Even worse, any aerosols that remained trapped inside the passive box tended to spill out into the room when the box was removed. These issues were confirmed by Hung et al. (2021), who observed that a sealed aerosol enclosure without active suction provided only minimal reduction in aerosols at the clinician’s head, and an unsealed design could even increase exposure at certain positions [31]. Collectively, such studies indicate that active ventilation or suction is necessary to make barrier enclosures truly effective [31]. Our findings are consistent with this concept: active HEPA-assisted extraction can improve aerosol control under the tested bench and exploration clinical conditions. This pattern is consistent with prior studies showing that barrier-plus-extraction strategies outperform barrier use alone [30,31].

Beyond acrylic boxes, other innovative barrier systems have been explored. Soft intubation tents or plastic sheets, for example, were introduced to improve maneuverability compared with rigid boxes. Low-cost clear plastic drapes were also explored as passive barriers and were shown in proof-of-concept testing to reduce droplet spray during extubation, although they did not provide active aerosol extraction [33]. In a Hong Kong study, an “intubation tent” allowed faster intubation times and fewer breaches than a hard plexiglass box [32]. However, this tent still relied on passive enclosure and did not incorporate active filtration [32]. Negative-pressure equipped enclosures like FNPACS combine the maneuverability advantages of flexible barrier systems with the added benefit of active aerosol extraction. Several teams have converged on similar concepts. Park et al. (2023) described a negative-pressure aerosol box with closed panels and vacuum assistance designed to function as a mini isolation chamber [5]. Another example is the negative-pressure isolation device reported by Shin et al. [5,34], which reduced aerosol exposure in a randomized controlled trial. Taken together, these studies support the view that sealed enclosures with active air extraction can improve protection during aerosol-generating procedures. Compared with other systems, a distinguishing feature of FNPACS is its foldable, lightweight construction, which further addresses logistical and ergonomic challenges noted with earlier devices.

### 4.3. Ergonomics, Usability, and Deployability Advantages

A critical design goal for FNPACS was to enhance protection without compromising procedural efficiency or operator safety. This consideration is particularly important because barrier devices have historically raised ergonomic concerns by restricting airway access, limiting hand movement, and complicating emergency maneuvers. In the present study, the usability findings showed that, after data cleaning, the system was generally well accepted while also revealing several clear priorities for refinement.

Forty-seven evaluable questionnaires were included in the final analysis after the exclusion of two non-discriminating response sets. Overall acceptance was high, particularly in domains related to perceived safety, learnability, ease of use, and physical acceptability. Participants generally found the system easy to learn and operate, and the results suggest that it can be incorporated into simulated clinical workflows without a substantial training burden.

Physical design features were also viewed favorably. Responses indicated broad acceptance of the device dimensions, chamber opening, weight, appearance, and post-use handling. Taken together, these findings suggest that the foldable chamber, lightweight structure, and compact footprint contributed meaningfully to user acceptance and bedside practicality. This emphasis on deployability is consistent with prospective clinical evaluation of a patient isolation hood, in which favorable staff acceptance and successful bedside implementation supported continued routine use [35].

Not all usability dimensions were equally strong. More mixed responses were seen for task speed, procedural control, recovery from mistakes, frequent future use, and ease of adjustment. These patterns suggest that, although the device was generally perceived as safe and usable, integration into routine workflow remains an important area for further improvement. In particular, the findings indicate that users did not uniformly experience the device as intuitive or unobtrusive during more demanding procedural tasks.

The qualitative feedback reinforced these quantitative patterns. Participants most often highlighted the HEPA filter, negative-pressure mechanism, foldable design, transparency, ease of assembly, and lightweight construction as key strengths. At the same time, the most common suggestions for improvement concerned the fastening system and access ergonomics. The Velcro-based sealing method was often perceived as insufficiently secure or cumbersome, and users also recommended improvements to hand-port design, assistant access, frame stability, overall weight, and adaptability to different clinical needs. Overall, the usability findings point to a clear direction for the next design iteration: stronger fastening and sealing, better hand-port ergonomics, improved structural stability, additional access options, lighter materials, and greater adaptability in future prototypes. For the next design iteration, we plan to evaluate alternatives to the Velcro-based seal, including gasket-assisted quick-lock interfaces and clamp- or magnetic-assisted closures, to improve seal repeatability during setup and removal. Frame refinement will focus on lighter but stiffer options, such as thin-wall aluminum tubing or reinforced engineering polymers, provided that foldability, visibility, and rapid emergency removal are preserved. These design changes will be assessed together with revised hand-port geometry and assistant-access openings to improve procedural ergonomics without compromising the negative-pressure airflow path.

Taken together, the usability evidence suggests that FNPACS already possesses several clinically meaningful ergonomic strengths: it is lightweight, portable, foldable, transparent, and generally easy to learn and use. These characteristics may offer practical advantages over heavier rigid barrier systems and support further evaluation of its deployability in clinical settings. At the same time, the user feedback makes clear that the next development cycle should prioritize improving seal reliability, hand-port ergonomics, assistant access, frame stability, and adaptability to different clinical scenarios. Accordingly, FNPACS should be presented not only as a functional negative-pressure containment device but also as a user-informed design platform with a clear and practical pathway toward broader clinical deployment.

## 5. Conclusions

This study provides preliminary evidence that FNPACS is a feasible near-source aerosol-control platform for aerosol-generating procedures. In bench testing, the system produced stable negative pressure, high HEPA filtration efficiency, and no detectable downstream breakthrough during PAO challenge. The DPM particle-tracking analysis further supported size-dependent aerosol-surrogate transport toward the HEPA pathway under HEPA-ON active-extraction conditions, while also indicating internal wall deposition as a secondary particle fate. The patient swab component should be interpreted as an exploratory, proof-of-concept localization study rather than a quantitative test of clinical viral containment efficiency. After separating control-limited cases from the primary containment interpretation, the clearest interpretable clinical finding was reproducible top-surface contamination under HEPA-OFF conditions for P10, whereas HEPA-ON findings were isolated, high-Ct, or not reproducibly confirmed. These findings support further development and controlled evaluation of FNPACS, but they do not establish definitive clinical containment efficacy.

Its portability, foldability, and generally favorable usability profile suggest potential for bedside use. The next development phase should include larger control-valid clinical studies, standardized aerosol or particle-based validation, and formal medical-device verification. Anticipated regulatory and testing steps include completion of electrical safety testing under IEC 60601-1 [36], continued electromagnetic compatibility documentation under IEC 60601-1-2 [22], risk-management documentation under ISO 14971 [37], usability engineering under IEC 62366-1 [38], biocompatibility assessment of patient-contacting materials under ISO 10993 [39] where applicable, and quality-management alignment with ISO 13485 [40]. These steps will help define the device’s eventual regulatory pathway and role in infection-control practice, consistent with broader bioengineering emphasis on safety, quality, and regulatory criteria during clinical translation [41].

## 6. Patents

A patent related to the work reported in this manuscript has been filed under patent application No. PI2020004291 and is currently pending.

## Figures and Tables

**Table 1:** Inflow velocity and differential pressure across the HEPA filter at different fan-speed settings.

Fan-Speed Setting (%)	Approximate Fan Speed (rpm)	Inflow Velocity at Prefilter Opening (m/s)	Differential Pressure Across HEPA Filter (Pa)
10	1330	0.18–0.20	−0.7
20	2600	0.38–0.40	−3
30	3990	0.62–0.64	−5
40	5320	1.02–1.12	−10
50	6650	1.14–1.18	−20
60	7980	1.92–1.94	−30
70	9310	2.04–2.28	−45
80	10,640	2.56–2.60	−60
90	11,970	2.70–2.78	−67

Note: Inflow velocity was measured at the center of the prefilter opening using a TSI 9515 hot-wire anemometer (VelociCalc 9515, TSI Incorporated, Shoreview, MN, USA). Differential pressure across the HEPA filter was measured using an independent pressure sensor across the filter. These measurements describe the prefilter-opening velocity and were not intended to quantify local in-chamber velocity changes with or without a patient-simulated occupant. The values are specific to the outlet/filter configuration tested in this prototype and may differ if the suction outlet position or geometry is changed.

**Table 2:** DPM particle outcome classification under HEPA-ON active negative-pressure conditions.

Particle Diameter	Number Tracked	Transported to HEPA Pathway (%)	Deposited on Internal Wall (%)
0.3 µm	1000	86.1	13.9
1 µm	1000	84.0	16.0
5 µm	1000	76.3	23.7
10 µm	1000	73.0	27.0
Total	4000	79.9	20.2

**Table 3:** The results of the main questions in user acceptance testing.

No	Questions	Strongly Agree	Agree	Somewhat Agree	Neutral	Somewhat Disagree	Disagree	Strongly Disagree
1	Confidence to Use	10 (21.3%)	16 (34.0%)	16 (34.0%)	5 (10.6%)	0.0	0.0	0.0
2	Feel Secure	10 (21.3%)	22 (46.8%)	10 (21.3%)	4 (8.5%)	1 (2.1%)	0.0	0.0
3	Device Size Reasonable	12 (25.5%)	19 (40.4%)	5 (10.6%)	8 (17.0%)	1 (2.1%)	2 (4.3%)	0.0
4	Chamber Opening Size	12 (25.5%)	18 (38.3%)	6 (12.8%)	6 (12.8%)	2 (4.3%)	3 (6.4%)	0.0
5	Device Weight Reasonable	12 (25.5%)	20 (42.6%)	11 (23.4%)	4 (8.5%)	0.0	0.0	0.0
6	Chamber Weight Reasonable	15 (31.9%)	21 (44.7%)	9 (19.1%)	2 (4.3%)	0.0	0.0	0.0
7	Disposal Ease	17 (36.2%)	16 (34.0%)	11 (23.4%)	2 (4.3%)	1 (2.1%)	0.0	0.0
8	Appearance Reasonable	16 (34.0%)	18 (38.3%)	12 (25.5%)	1 (2.1%)	0.0	0.0	0.0
9	Like to Use Frequently	8 (17.0%)	13 (27.7%)	11 (23.4%)	12 (25.5%)	3 (6.4%)	0.0	0.0
10	Easy to Adjust	10 (21.3%)	16 (34.0%)	13 (27.7%)	7 (14.9%)	1 (2.1%)	0.0	0.0

**Table 4:** Clinical trial outcomes for the device, tested on 11 patients at 6 different sampling positions.

Sample	Petri Dish (Positive Control)	Oral Cavities (Positive Control)	A (Top)		B (Front)		F (Filter-Outlet Exterior Surface)		Other Positions (C, D, E)		Use in Primary Interpretation	Overall Interpretation
			HEPA OFF	HEPA ON	HEPA OFF	HEPA ON	Control	Post- Cough	HEPA OFF	HEPA ON		
P1	NA	NA	N	N	N	N	N	N	N	N	Descriptive only—assay positive control only.	Descriptive only; all environmental swabs were negative, with assay positive control only; not used to infer containment efficiency.
P2	N	NA	N	N	N	IP	N	N	N	N	No—control-limited/inconclusive; descriptive only.	Control-limited/inconclusive; isolated front-site signal (Ct 37.79) with non-supportive patient controls; excluded from primary containment interpretation.
P3	PP	NA	N	N	N	N	N	N	N	N	No—control-limited/inconclusive; descriptive only.	Control-limited/inconclusive; no environmental detection but patient controls were non-supportive; excluded from primary containment interpretation.
P4	N	NA	N	N	PP	PP	N	N	N	N	No—control-limited/inconclusive; descriptive only.	Control-limited/inconclusive; presumptive front-site signals (Ct 37.82 OFF; Ct 37.81 ON) with non-supportive patient controls; excluded from primary containment interpretation.
P5	N	NA	N	N	PP	N	N	N	N	N	No—control-limited/inconclusive; descriptive only.	Control-limited/inconclusive; presumptive front-site signal (Ct 37.99) with non-supportive patient controls; excluded from primary containment interpretation.
P6	N	NA	N	N	N	IP	N	N	N	N	No—control-limited/inconclusive; descriptive only.	Control-limited/inconclusive; isolated front-site signal (Ct 38.57) with non-supportive patient controls; excluded from primary containment interpretation.
P7	N	NA	N	N	N	N	N	N	N	N	No—control-limited/inconclusive; descriptive only.	Control-limited/inconclusive; no environmental detection but patient controls were non-supportive; excluded from primary containment interpretation.
P8	N	NA	N	N	N	N	N	N	N	N	No—control-limited/inconclusive; descriptive only.	Control-limited/inconclusive; no environmental detection but patient controls were non-supportive; excluded from primary containment interpretation.
P9	N	P	N	N	N	IP	N	N	N	N	Descriptive only—isolated high-Ct signal; not reproducibly confirmed.	Descriptive only; isolated high-Ct front-site signal under HEPA ON (Ct 38.34); not reproducibly confirmed.
P10	P	P	P	N	N	N	N	PP	N	N	Yes—control-valid and reproducible HEPA-OFF top-surface signal.	Control-valid; reproducible HEPA-OFF top-surface positive finding; included in primary interpretation. HEPA-ON outlet-associated E-gene-only signal was isolated and not reproducibly confirmed.
P11	N	P	N	N	PP	PP	PP	N	N	N	Descriptive only—isolated presumptive signals; not reproducibly confirmed.	Descriptive only; no reproducible environmental positive; isolated presumptive signals only (Ct 37.79, 37.75, 39.37); not used to infer containment efficiency.

NA, not available. N, negative. P, confirmed positive. IP, isolated positive signal detected in an individual assay output but not reproducibly confirmed across replicates. PP, presumptively positive (E-gene-only signal without reproducible confirmation). A represents the top surface, B the front panel, C the left side, D the tight side, E the back panel, and F the filter-outlet exterior surface. C, D, and E are pooled as Other Positions (C, D, E). The “Use in primary interpretation” column indicates whether each case was included in the primary interpretation of the clinical swab findings. Control-limited/inconclusive cases were retained for transparency but were excluded from the primary interpretation because their Petri dish and/or oral-cavity controls were negative, unavailable, or otherwise non-supportive.

## Data Availability

The data supporting the findings of this study are available from the corresponding author upon reasonable request. Due to privacy and ethical restrictions associated with the clinical swab study involving human participants, individual-level clinical data are not publicly available. De-identified data may be made available on reasonable request, subject to institutional approval.

## Abbreviations

The following abbreviations are used in this manuscript:

AGP	Aerosol-generating procedure
AGPs	Aerosol-generating procedures
CFD	Computational fluid dynamics
Ct	Cycle threshold
FNPACS	Foldable negative-pressure aerosol-containment system
HCW	Healthcare worker
HCWs	Healthcare workers
HEPA	High-efficiency particulate air
IEC	International Electrotechnical Commission
ISO	International Organization for Standardization
MREC	Medical Research Ethics Committee
NEBB	National Environmental Balancing Bureau
PAO	Polyalphaolefin
PPE	Personal protective equipment
PWM	Pulse-width modulation
RNA	Ribonucleic acid
rRT-PCR	Real-time reverse-transcription polymerase chain reaction
SARS-CoV-2	Severe acute respiratory syndrome coronavirus 2
SD	Standard deviation
SST	Shear stress transport
